# Integrative modeling of JCVI-Syn3A nucleoids with a modular approach

**DOI:** 10.1016/j.crstbi.2023.100121

**Published:** 2023-12-22

**Authors:** David S. Goodsell, Ludovic Autin

**Affiliations:** a Department of Integrative Structural and Computational Biology, The Scripps Research Institute, La Jolla, CA, 92037, USA; b Research Collaboratory for Structural Bioinformatics Protein Data and Institute for Quantitative Biomedicine, Rutgers, The State University of New Jersey, Piscataway, NJ, 08854, USA

**Keywords:** Whole-cell computational model, Minimal genome cell, Bacterial nucleoid

## Abstract

A lattice-based method is presented for creating 3D models of entire bacterial nucleoids integrating ultrastructural information cryoelectron tomography, genomic and proteomic data, and experimental atomic structures of biomolecules and assemblies. The method is used to generate models of the minimal genome bacterium JCVI-Syn3A, producing a series of models that test hypotheses about transcription, condensation, and overall distribution of the genome. Lattice models are also used to generate atomic models of an entire JCVI-Syn3A cell.

## Highlights

• Models of bacterial nucleoids are built with a new modular construction approach.
• Nucleoid models of minimal cell JCVI-syn3A integrate available experimental data.
• Hypotheses for placement of RNA polymerases and cohesins are explored.

## Graphical abstract

**Figure:**
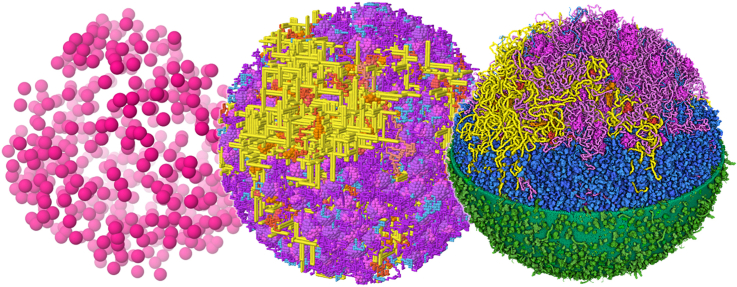

## 1 Introduction

Nucleoids present one of the most difficult challenges for 3D modeling of entire bacterial cells. Models must integrate knowledge at multiple scale levels, from local molecular interactions to large-scale cellular ultrastructure. At the sequence level, the genome encodes position-specific information. At the molecular level, regions of the genome form functional interactions with multiple systems involved in transcription, replication, and regulation. At the whole-genome level, multiple mechanisms are employed for compacting the DNA and packing it within the limited space of the cell. In addition, DNA and associated molecules are intimately linked to related processes, such as translation by ribosomes and folding and transport of nascent proteins.

Many labs are approaching the modeling of bacterial nucleoids at various levels of detail. A full review of the field is beyond the scope of this report, but few highlights are described here. Systems-based approaches typically focus on the information content of the cell and its expression, as exemplified in a Whole Cell model of mycoplasma that follows the states of the nucleoid across the entire cell cycle (Karr et al., 2012). Structure-based approaches typically employ some form of coarse-graining, given the large size of bacterial genomes. A wide range of coarse-graining is employed, such as modeling the entire *Escherichia coli* genome with 200–1400 beads to explore the consequences of double strand breaks (Dorier and Stasiak, 2013); a highly branched model that represents plectonemes with single strings of beads separated by 200 nm (Mondal et al., 2011), or a 20 bp/bead molecular dynamics study to explore the consequences of fis protein binding (Koşar et al., 2022). Coarse-grain models of nucleoids are also generated directly from experimental contact data, such as the models of *Caulobacter crescentus* with beads representing restriction fragments (Umbarger et al., 2011) using IMP (Russel et al., 2012), and the 5 kb/bead representation of the *Escherichia coli* genome (Lioy et al., 2018) generated with ShRec3d (Lesne et al., 2014).

Atomic and near-atomic models of entire genomes are still a rarity. Pioneering studies include a 1 nucleotide/bead model of *Escherichia coli* based on transcriptional frequencies (Hacker et al., 2017) and a 15 bp/bead model of *Caulobacter crescentus* based on HiC data which was then expanded to the atomic level by assuming ideal properties for the DNA double helix (Yildirim and Feig, 2018). We used a similar progressive-refinement approach in our own work (Goodsell et al., 2018), generating 10bp/bead models with a lattice-based approach, optimizing them off lattice, and using them as a scaffold for building a full atomic model in CellPACKgpu (Maritan et al., 2022). This approach was expanded in the context of Lattice Microbe simulations to embed a nucleoid model within a cryoEM tomogram of JCVI-syn3A (Gilbert et al., 2021), and later further enhanced as a 10 bp/bead model used in Brownian dynamics simulations of the genome and DNA (Gilbert et al., 2023). The latter study also included a forward-looking simulation of genome segregation using a reduced system of roughly 1/10 the size of JCVI-syn3A. In addition, these models have been used to generate models compatible with the Martini force field, opening the door to whole-cell molecular dynamics simulations of this synthetic organism (Gilbert et al., 2023; Stevens et al., 2023). In addition, a rapid parallel algorithm for generation of large nucleic acid structures was presented for use in interactive visualization environments (Klein et al., 2019).

In our previous work, we developed a lattice-based method for the generation of 3D coarse-grain structural models of entire bacterial genomes. These models are parameterized with one bead per ten base pairs and include user-defined superhelical plectonemes and coarse-grain representations of nucleoid associated proteins. The previous implementation was largely hardwired for a single circular genome (Goodsell et al., 2018) with spherical representations for nucleoid-associated proteins and coarse-grain messenger RNA (Maritan et al., 2022). In this report, we generalize this work with a modular implementation of the method that allows incorporation of cryoEM data and streamlines description and modeling of more complex topologies of component nucleic acids and proteins. We present the basic method and apply it to one of the simplest genomes of a free-living organism: the minimal genome cell JCVI-Syn3A.

The minimal-genome cell JCVI-Syn3A was created with a synthetic genome containing essential genes from *Mycoplasma mycoides*, reducing the number of genes from 901 to 493, making it the organism with the smallest genome that can be grown in laboratory culture (Breuer et al., 2019; Hutchison et al., 2016). The simplicity of JCVI-Syn3A has made it an attractive subject for research. The integrative modeling presented here relies on extensive experimental data and modeling work, including the genome, proteome, HiC data, EM tomograms of whole cells, and abundant biochemical studies. In particular, we rely on two recent modeling studies for many of the parameters used to define the nucleoid models presented here (Gilbert et al., 2021; Stevens et al., 2023), and build on our previous illustrative work on this cell (Goodsell, 2022).

## 2 Methods

### 2.1 Overview

**Fig. 1:**
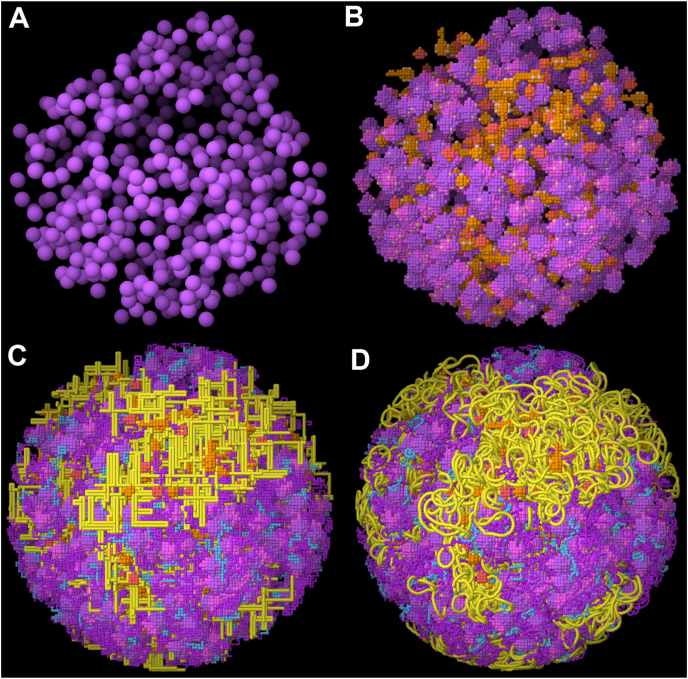
Overview of the method. (A) Ribosome positions are taken from cryoEM tomograms. (B) Coarse-grain masks of ribosomes (purple) are placed in random orientations at the experimentally-determined positions, then RNA polymerase and SMC positions (orange) are generated programmatically. (C) Lattice models of RNA (purple), DNA (yellow), and nascent protein (blue) chains connect to control points on the molecules. (D) Chains are optimized off lattice. Most of the images in this report were created with Jmol (Hanson, 2010). (For interpretation of the references to color in this figure legend, the reader is referred to the Web version of this article.)

ModularLattice is built based on several design decisions (Fig. 1). Molecules are represented as lattice-based models, called “molecular masks” here, based on experimental atomic structures. Currently, three types of molecules are implemented: RNA polymerase, ribosome, and SMC (structural maintenance of chromosomes) cohesins. These molecular masks are placed in fixed positions within the lattice, with one of 24 possible 90° rotations. The positions may be obtained from cryoEM structures (such as ribosomes) or generated programmatically (currently, polymerase and SMC). These mask positions are then connected by segments of DNA, RNA or disordered protein to yield the final nucleoid model, for example, with DNA chains that connect all the RNA polymerase positions into a single DNA circle. Molecular masks include control points that define the sites of attachment for the chain segments. For example, the RNA polymerase mask has two control points for the 5′ and 3′ ends of the DNA, and a third control point for the 3’ end of the nascent RNA. Chain segments connect these control points with two methods: a point-to-point connection between two control points, or a random walk from one control point. Three types of chain segments are currently implemented: DNA (with user-defined supercoiling), RNA, and disordered protein. Finally, the model is optimized off-lattice with constraints for connectivity, steric occlusion, and fiber stiffness.

### 2.2 Molecular masks of nucleoid-related molecules

**Fig. 2:**
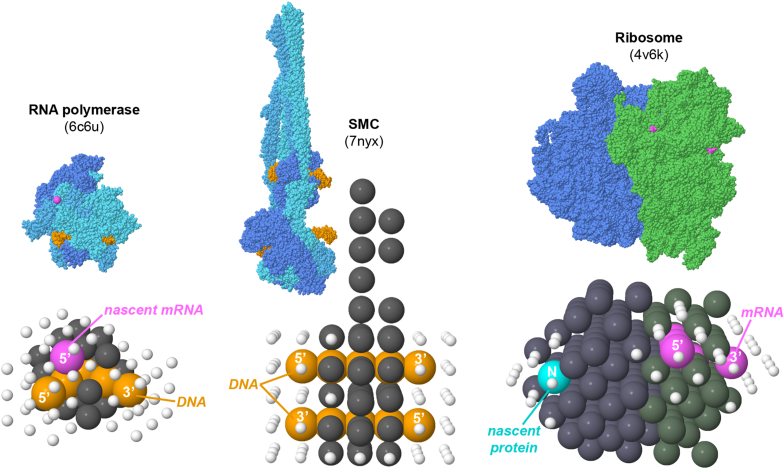
Molecular masks. Atomic structures are shown at the top and lattice-based models are shown at the bottom. Molecule points are shown in dark gray. DNA points in orange, RNA points in magenta, and nascent protein points in turquoise, all with control points labeled as 5′, 3′ or N termini. Insulating points are shown with smaller spheres in white. (For interpretation of the references to color in this figure legend, the reader is referred to the Web version of this article.)

Lattice-based models, called “molecular masks” here, of RNA polymerase, ribosome, and SMC are generated by centering the molecule on the origin, embedding it in a grid with 3.4 nm spacing, and choosing points within one grid spacing of an atom (Fig. 2). Points representing the DNA/RNA/protein chains and control points are then assigned manually. After assigning molecule points and chain points, all free points surrounding each control point are assigned as “insulating” points and added to the mask. These insulating points ensure that chains from neighboring molecules do not occlude control points and block construction of a chain from the control point. Finally, two additional molecule points were added manually to SMC at the point between neighboring 5′ and 3’ ends; these are intended to block construction of SMC loops that are only one step long.

The current implementation includes three molecules: a RNA polymerase elongation complex from PDB ID 6c6u, a ribosome elongation complex from PDB ID 4v6k, and SMC is modeled using the structure of MukBEF with two DNA oligonucleotides, PDB ID 7nyx.

### 2.3 Placing molecular masks in CryoEM tomograms

The central goal of this work is to incorporate information from cryoEM tomography in whole-cell structural models. Typically, only a subset of the nucleoid-related molecules will be visible. In this step of the nucleoid-generation pipeline, molecules with experimentally-determined locations are placed in their known positions, and the remaining molecules are placed based on biochemical information and hypotheses about distribution and connectivity. In the case of JCVI-syn3A, ribosomes can be clearly identified and placed, and RNA polymerase and SMC positions are generated programmatically.

Ribosome positions were kindly provided by Benjamin R. Gilbert, Vinson Lam, Elizabeth Villa, and Zaida Luthey-Schulten (Gilbert et al., 2021). They are based on cryoEM segmentation of their “small” cell, which they then transformed to a spherical cell with radius 201 nm (Fig. 1A). Ribosome masks are placed at each position with random orientations chosen from the 24 possible 90° rotations. The model includes 503 ribosomes. Some of the experimental positions show small clashes with neighbors, which are resolved by jittering ribosomes in the local neighborhood as the lattice-based models are placed.

**Fig. 3:**
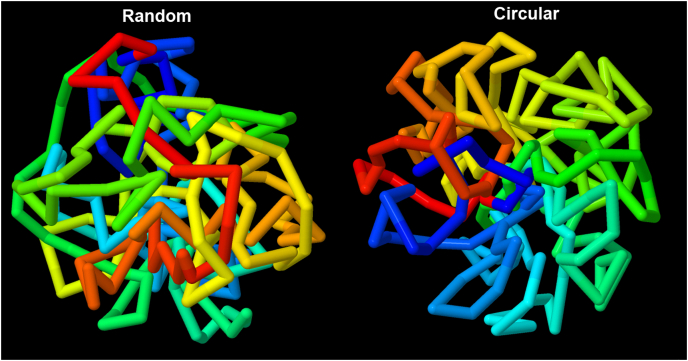
Placement of RNA polymerase. The “Random” approach used a random walk throughout the entire cell interior and the “Circular” approach limits placement to a rectangular box that rotates around a randomly-placed axis. In this illustration, lines are drawn between polymerase positions with rainbow coloring from origin around the circle back to the origin.

Polymerases are then placed in the free spaces with a coarse random walk (Fig. 3). The first polymerase is placed randomly, and subsequent polymerases are placed with random position and orientation at a distance less than 20 nm from the previous position. In the current implementation, the entire random walk is (wastefully) performed multiple times until a path is found that provides closure of the DNA circle. The current model includes 187 RNA polymerases (Gilbert et al., 2021), all of which are treated as actively elongating.

Finally, SMC positions are placed between the polymerase positions. For simplicity, the model includes 187 SMC molecules, which is close to the value of 202 reported for the proteome (Gilbert et al., 2021). SMC are centered on the line between successive polymerase positions, then jittered to find a nearby free lattice position.

A second hypothesis for polymerase placement was also implemented, to demonstrate the flexibility of the method (Fig. 3). This generates a circular structure for the overall genome, as has been observed in many bacteria. A random rotational axis is chosen that passes through the cell center, and a rectangular box is aligned with the axis, offset such that one slice of the cell is enclosed. Polymerases are placed randomly within the box, and the box is rotated by one revolution over the course of placing the whole genome.

### 2.4 Defining connectivity

A “.connect” file defines how to connect control points with DNA/RNA/protein segments. To simplify generation and output of easily-parsed model coordinates, this file is designed to specify full, continuous chains. For example, it will define all of the segments, in order, needed to build a circular DNA with many polymerases and SMC. The short snippet below specifies the creation of a mRNA strand with one ribosome in the center. In the final model, coordinates for this mRNA will include 48 beads for the first segment, followed by 5 beads that are included in the molecular mask for the ribosome, followed by 48 beads for the second segment. A full description of the format is included in the documentation.

**Figure:**
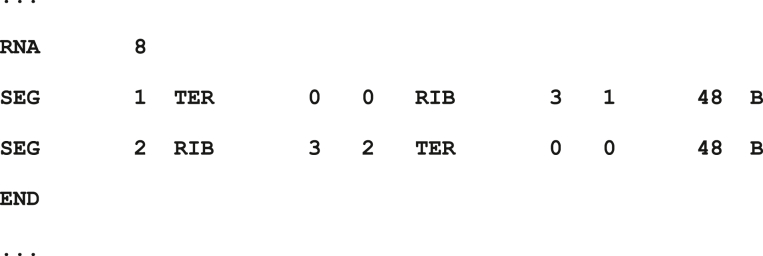

**Fig. 4:**
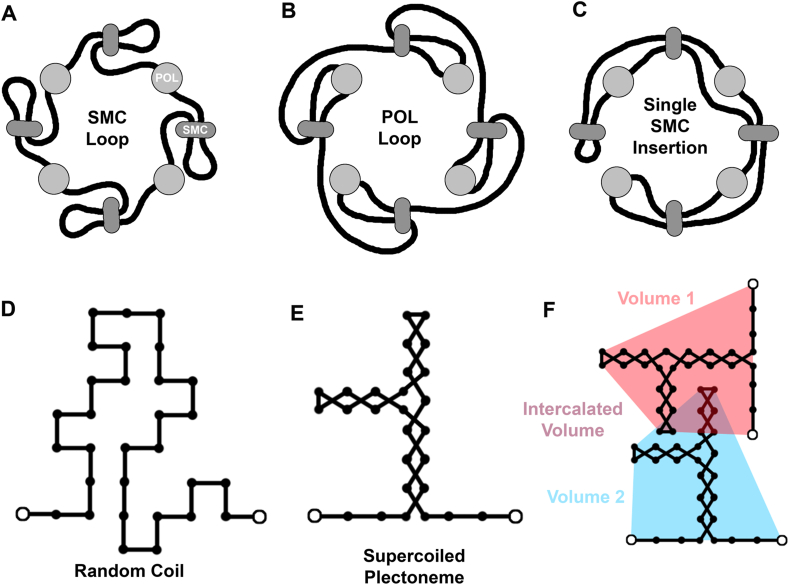
(A–C) Three idealized models of polymerase/SMC/DNA connectivity explored in this study. Each DNA segment is treated as either (D) a relaxed random coil or (E) a superhelical plectoneme. Control points for polymerase or SMC are shown as circles filled with white. (F) Schematic of the method used to calculate volumes for each transcription unit, and the intercalated volume of overlap between different volumes.

In the current study, we explored three different connectivities for the circular genome of JCVI-syn3A that incorporate different hypotheses for the role of SMC (Fig. 4A–C). The “SMC loop” model places SMC between transcribing polymerases, with an extruded loop of DNA. The “POL loop” model places transcribing polymerases within the extruded DNA loop. The “Single SMC insertion” model postulates a single site of insertion for SMC, with subsequent diffusion of SMC along the DNA, as seen in organisms such as *Bacillus subtilis* (Wang et al., 2017).

**Fig. 5:**
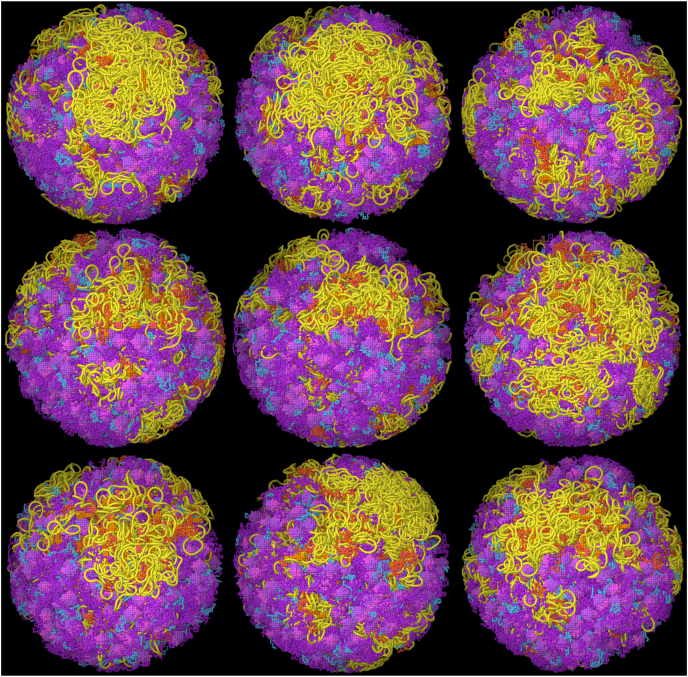
Nine instances of the JCVI-syn3A model, shown from the same viewpoint.

**Fig. 6:**
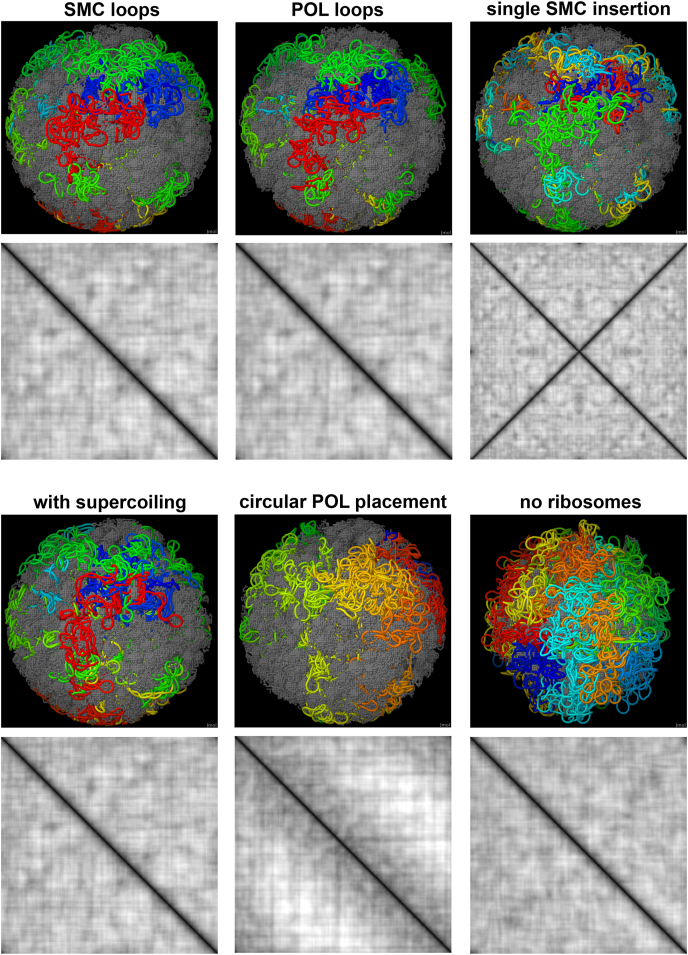
Representative instances of six variations on idealized models, with DNA colored by a rainbow and all other molecules colored gray. Plots show distances between the 187 transcription units, with close distances (primarily along the diagonal) in darker shades. Plots were calculated using 20 instances of each variation. (For interpretation of the references to color in this figure legend, the reader is referred to the Web version of this article.)

**Fig. 7:**
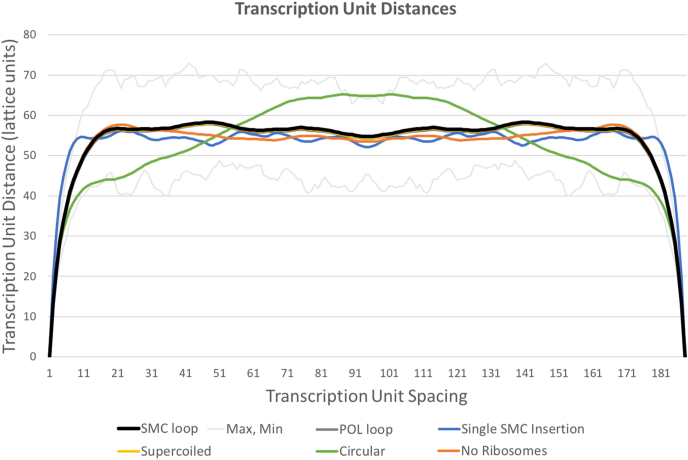
Analysis of the distance plots in Fig. 6, averaging all transcription unit distances for a particular spacing of transcription units in the genome (i.e., averaging values that are a constant horizontal/vertical distance from the diagonal). Max and min values are shown in light gray only for the default SMC loop model. Lattice units are 3.4 nm.

### 2.5 Generation of lattice models

ModularLattice reads the coordinate file of molecular masks and the connectivity file, and generates a lattice model for all of the connecting segments. Currently, nucleoid models are built in three steps: 1) a biased random walk connects segments with two control points; 2) these connected segments are filled out with either plectonemes or looped extensions to give the specified length; 3) segments with one control point are built using a random walk.

A biased random walk is used to connect two control points. At each step, the six possible step directions are tried randomly. If the step is closer to the target control point, it is immediately accepted. If not, the direction with the fewest occupied neighboring points is chosen. If the random walk has reached a dead end, the entire walk is restarted from scratch and repeated until a full path is obtained. The random walk is also modified with a chain-specific persistence length that automatically tries the direction of the previous step and accepts it if it is unoccupied. The number of these same-direction steps is controlled by a persistence parameter, set to 10 steps for DNA and 1 step (no bias) for RNA and protein in the current study. Random walks with one control point are performed similarly, starting from a control point and extending in random directions modified by the same persistence parameter.

The initial distances between two control points are typically shorter than the desired chain length, so two methods are provided for filling in missing points. The first replaces pairs of points in the chain with small orthogonal loops with lengths controlled by the persistence parameter. Loops are added at random positions in the growing chain until the desired length is reached. The result is a fractal-like randomly coiled chain that connects the two points, and represents a possible conformation in the absence of extensive supercoiling (Fig. 4D).

The second approach models a DNA segment that is supercoiled. Supercoiling induces formation of branched superhelices, termed plectonemes. Currently, a single branched plectoneme of appropriate length is inserted at the center of the connecting segment (Fig. 4E). Plectonemes are generated as in previous work (Goodsell et al., 2018) by first generating an on-lattice random walk from the insertion point and then splitting the random walk chain off-lattice into a superhelical path. Branching is based on an EM study of supercoiled plasmids (Boles et al., 1990), with branch lengths of ∼1000 bp. Currently, for long plectonemes, a central stem superhelix is created first, then branches are created alternately on the leading and lagging segments of the stem superhelix.

### 2.6 Off-lattice optimization

Optimization of lattice models uses an approach similar to previous work (Goodsell et al., 2018). To preserve the experimental cryoEM positioning of molecules, only portions of chains that are added in the lattice generation step are optimized off lattice; the mask positions for ribosomes, polymerases and SMC are frozen in place. As described previously, the optimization method includes simple constraints for bond lengths, non-bonded contacts, n-to-n+6 constraints for DNA persistence, and a spherical boundary that models the cell membrane.

### 2.7 Idealized and genome-based models

Two conceptions of the location of polymerase and SMC were explored in this study: an idealized model and a genome-based model. All models were created with a coarse grain representation at 10 bp/bead. The idealized model spaced polymerase and SMC equally throughout the genome, with equal-length DNA segments for all polymerase-SMC and SMC-SMC connections and a constant length of 91 beads for mRNA. Ribosomes were centered on a mature mRNA of length 101 beads, with 16 beads for nascent protein.

The genome-based model is based on GenBank: CP016816.2. The model includes all non-coding RNA and mRNA that encodes proteins with highest abundance in the proteome. Polymerases were placed at the center of the 187 most highly transcribed genes, with nascent mRNA of half gene length. Very short DNA segments are currently difficult to model with the lattice method, so neighboring genes where the centers are less than 900 bp were manually removed. SMC loops were assigned for segments longer than 230 beads, with lengths of 90 DNA beads between polymerase and SMC, and the remaining DNA assigned to a random loop extruded by SMC. For shorter DNA segments, SMC was centered on the segment using only the first DNA strand of SMC mask, modeling the complex before loop extrusion.

### 2.8 Draft atomic model of JCVI-syn3A incorporating the genome model

**Fig. 8:**
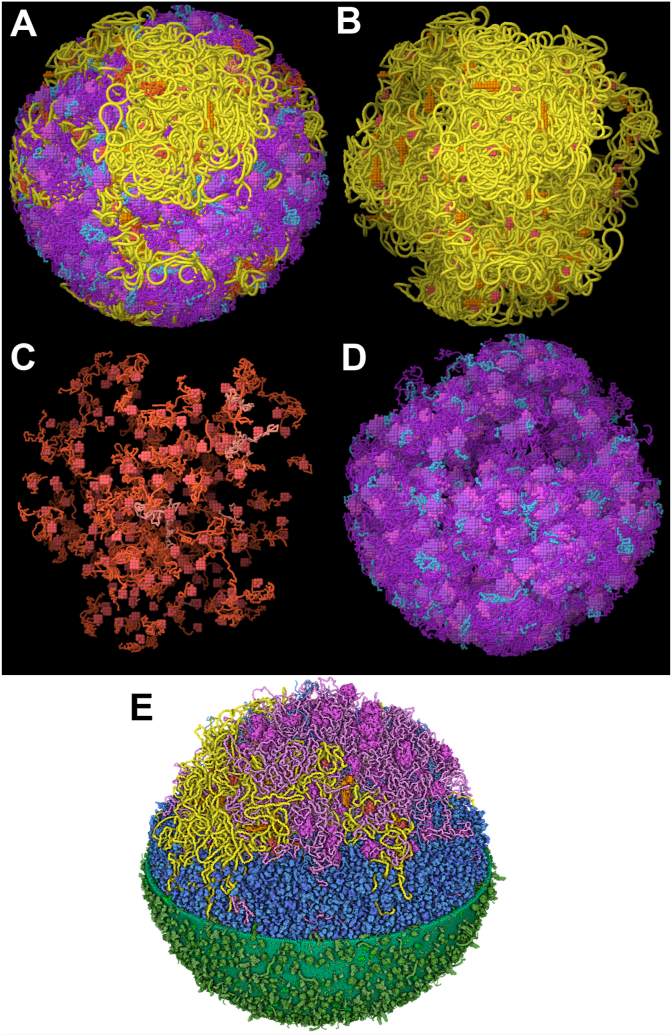
Tour of the JCVI-Syn3A nucleoid. 3D whole cell models are useful in non-expert settings to help viewers conceptualize the scale and complexity of biological molecules in their cellular context. These images show: (A) the entire nucleoid model, (B) the genome and associated polymerase and SMC proteins, (C) polymerases transcribing nascent RNA, with protein-coding mRNA in orange and non-coding RNA in lighter orange, and (D) ribosomes translating mature mRNA into proteins. (E) A draft atomic model of the entire cell includes the nucleoid model (magenta and yellow), soluble proteins and tRNA (blue), and membrane and membrane-bound proteins (green). (For interpretation of the references to color in this figure legend, the reader is referred to the Web version of this article.)

Building on previous work, we incorporated the genome-based model into a draft atomic model of all macromolecules in the JCVI-syn3A cell. The model is created similarly to a previous model of *Mycoplasma genitalium* (Maritan et al., 2022). Briefly, atomic structures for the proteome were gathered and curated in Mesoscope (Autin et al., 2020). As described in our previous study (Goodsell, 2022), we adapted the structural proteome used in the Mycoplasma model for proteins with obvious homologs, and predicted structures for proteins with unknown function using AlphaFold2 (Jumper et al., 2021). Models were generated interactively using CellPACKgpu (Klein et al., 2018), and optimized using NVIDIA FleX (Macklin et al., 2014) while keeping ribosomes, polymerases and SMC frozen. Finally, the model is visualized in real time in CellPACKgpu using the cellVIEW rendering technique (Muzic et al., 2015). For the model depicted in Fig. 8E, we use atomic radii for membrane and soluble macromolecules, and a slightly expanded radius of 3.0 Å for fibrous DNA, RNA and nascent protein to enhance visibility.

### 2.9 Analysis

Models were largely visualized with the stand-alone version of Jmol (Hanson, 2010), which allows nimble interactive exploration of different selection, representation, and coloring parameters with its natural language interface. Model features were analyzed by estimating the size and interactions of “transcription units.” Transcription units were defined as the segments of DNA connecting two successive polymerases in the model. Distance plots were calculated between the centroids of transcription units. Volumes and interdigitation of transcription units was estimated using a simple approach similar to a convex hull (Fig. 4F). The optimized model was embedded into a 3.4 nm grid; lines were drawn connecting all pairs of DNA beads in the transcription unit, and all nearest points along the line were assigned as part of the enclosed volume. Beads from other transcription units that fell within the enclosed volume were tallied to give an impression of the intercalation of neighboring units.

## 3 Results

### 3.1 Integrative models of JCVI-Syn3A

Fig. 5 presents nine instances of an integrative model of the JCVI-Syn3A nucleoid and ribosomes based on experimental location of ribosomes and genomic information. The large variability is the result of different orientations of ribosomes at the experimental positions and differences in polymerase and SMC placement. Note however that all instances show a large region filled with DNA just above center in this view. This is the result of a relatively ribosome-free region in the experimental tomogram.

One of the design goals of this project is to provide a method that allows rapid specification and construction of these types of models, to allow exploration of variability and different hypotheses. The modular approach, which requires only definition of mask positions and two connection methods, allows a wide variety of topologies to be specified and built. For construction, the current unoptimized pipeline requires roughly 20 min of computational effort on an iMac computer per instance. Most of this time is spent in the optimization step; the lattice-based modeling step is completed in several seconds. Due to the crowded nature of these environments, the current implementation of lattice generation fails roughly half of the time at various steps of connection, extension, and addition of tethered chains. In these cases, one or two repeats with a different random number seed consistently yielded a model. In addition, the current pipeline, which places polymerases randomly in the cytosol, fails in cases where polymerases are closely spaced along the sequence. As described in the Methods, this required manual intervention when choosing genes that would host polymerases in the current model.

### 3.2 Testing hypotheses with idealized models

Idealized models may be used to minimize changeable parameters when exploring hypotheses about structure and function. We used idealized models of Syn3A to explore the structural consequences of different hypotheses for the placement of polymerases and the genome in experimental tomograms. These models space polymerases evenly throughout the genome and use a consistent length for mRNA and nascent protein chains. Six variations were tested:

● SMC loop model. This is the default model that is most consistent with interpretations from the original report of the tomogram (Gilbert et al., 2021), with random placement of polymerases, loops formed by SMC, and no supercoiling.
● POL loop model. This model places polymerase within the loops extruded by SMC.
● Single SMC insertion. SMC binds at one point in the genome (chosen as the origin here) and diffuses along the chain.
● SMC loop with supercoiling. Same as the default SMC loop model, but with superhelical plectonemes in all DNA segments.
● Circular POL placement. Polymerases are placed to form a rough circle around an arbitrary axis.
● No ribosomes. This model creates the nucleoid in an empty cell free of ribosomes.

These models incorporate aspects of genome structure from various organisms. The SMC loop and POL loop models represent two extremes that result from random insertion of SMC, which is likely found in many bacteria, and the single SMC insertion model is an alternate arrangement that has been observed in specific organisms, such as *Bacillus subtilis (**Wang* et al.*, 2017**)*. The effect of supercoiling is studied by comparing the first and fourth variations. The circular POL placement model incorporates the observation that many bacterial genomes adopt a donut-like shape within the cell, for example, as observed for *Mycoplasma pneumoniae (**Trussart* et al.*, 2017**)*. Finally, the no ribosome model is included as a control. In addition, these models are intended to evaluate the utility and flexibility of the modular modeling method.

For each variant, twenty instances were generated. For each instance, ribosomes were placed in random orientations on experimental positions and new polymerase and SMC positions were generated. When possible, identical polymerase/SMC placement was used across variants. For example, instance 1 of the SMC loop, POL loop, single SMC insertion, and supercoiled models all use the same ribosome/polymerase/SMC placement, but have different connectivities that are specific to the models.

The models were analyzed in several ways. For manual inspection of genome features, a representation was used with DNA colored with rainbow colors. For example, the first instance for each variant is depicted in Fig. 6. To quantify features seen in these visualizations, we focused on the properties of “transcription units,” which we define as the stretch of DNA between successive polymerase transcription sites. Analysis of the volume of the 187 transcription units is included in Table 1 and a distance plot between the centroids of the transcription units is shown in Fig. 6.

**Table 1:** Volume of transcription units.

	Volume	Intercalators	Percent Intercalators
No supercoiling, random POL			
SMC loop	4882 (73)	283 (13)	5.8%
POL loop	5478 (83)	362 (15)	6.7%
Single SMC insertion	5988 (114)	422 (16)	7.0%

SMC loop with supercoiling	6113 (134)	377 (17)	6.2%
SMC loop with circular POL	4894 (68)	303 (14)	6.2%
SMC loop with no ribosomes	5332 (81)	255 (10)	4.8%

“Volume” is calculated with an approximation of the convex hull and “Intercalators” are beads from other transcription units that fall within the volume. Values evaluated with 20 models, with standard deviations in parentheses.

In the visualization, SMC loop, POL loop, and supercoiled models are very similar, with the supercoiled model showing slightly more extension of DNA loops. The single SMC insertion model shows similar overall placement of DNA, primarily in the larger voids between ribosomes, but the identity of the DNA is different, with distant parts of the DNA circle brought into close proximity (for example, notice the yellow and cyan regions that are closely opposed near the top of the image). Unsurprisingly, the circular POL model appears quite different since the polymerases are placed with an entirely different hypothesis. The ribosome-free model is a tangle of DNA uniformly filling the cytosol space.

Looking at the transcription unit volumes in Table 1, we quantify some of these differences. The SMC loop model with either random or circular POL placement both have the smallest volumes for transcription units, and the least intercalation by neighboring transcription units. Supercoiling and single SMC insertion have the largest volumes and the most intercalation of neighbors, both the result of the more extended nature of the transcription units in these variants. The ribosome-free model shows the crowding effect of ribosomes--by comparing the SMC loop and ribosome-free volumes, we see that the effect is surprisingly small.

Looking at the distances between transcription unit centroids (Fig. 6, Fig. 7), the trends are similar. SMC loop, POL loop and supercoiled models are almost identical, with largely featureless plots off the diagonal and superimposable traces in the average distance plot. The single SMC insertion variant has a narrower diagonal, showing that transcription units have fewer close contacts with their immediate neighbors than are seen with other variants, and the characteristic cross-diagonal that is expected for this type of SMC interaction. The circular POL placement models show the opposite trend, with more close contacts with neighbors and fewer cross-circle contacts.

## 4 Discussion

We envision that these types of 3D whole cell models will be useful in two different applications. First, they may be used as research tools to explore hypotheses about the structure of components that are not readily visible in experimental tomograms. For example, in the idealized nucleoids analyzed here, the SMC loop, POL loop, and supercoiled results are all virtually identical in simulated distance maps, and are consistent with the largely featureless experimental HiC data, as seen in Fig. 7 of (Gilbert et al., 2021). Surprisingly, the ribosome-free model is also similar to the full models.

These types of models have also shown great utility in science outreach and education, to help non-expert viewers gain a conceptual understanding of the crowded and complex nature of living cells. JCVI-syn3A is an ideal system for this, given its relative simplicity and strong place in the foundational studies of the molecular basis of life. For example, Fig. 8 presents a series of images that present a tour of the cell model. This allows viewers to appreciate the amount of DNA, the number of polymerases and ribosomes, and the level of coordination that is needed to support life of this cell.

This integrative model represents a step towards a comprehensive 3D conception of a JCVI-syn3A nucleoid and entire cell. The modular construction approach presented here allows nimble extension of additional molecular features without the need to create multiple new methods to model each one. For example, addition of expressomes and membrane-bound ribosomes is a simple matter of creating templates and placing them appropriately, and higher-order structures like polysomes and replication intermediates simply require additional bookkeeping in the specification of connectivities.

## CRediT authorship contribution statement

**David S. Goodsell:** Conceptualization, Methodology, Software, Investigation, Data curation, Writing – original draft, Visualization. **Ludovic Autin:** Conceptualization, Methodology, Software, Investigation, Data curation, Writing – original draft, Visualization.

## Declaration of competing interest

The authors declare the following financial interests/personal relationships which may be considered as potential competing interests: David S. Goodsell reports financial support was provided by National Institutes of Health. If there are other authors, they declare that they have no known competing financial interests or personal relationships that could have appeared to influence the work reported in this paper.

## Data Availability

Source code, modeling pipeline script, Jmol visualization script, and representative lattice, optimized and atomic models are freely available at https://github.com/ccsb-scripps/ModularNucleoid.
